# Supplementary material for: High Light Acclimation Mechanisms Deficient in a PsbS-Knockout Arabidopsis Mutant
Source: Int J Mol Sci. 2022 Feb 28;23(5):2695. doi: 10.3390/ijms23052695 (PMC8910700; doi:10.3390/ijms23052695)
Supplement: Supplementary file 1 [file ijms-23-02695-s001.zip › ijms-1587383-supplementary.pdf]

Supplemental Table S1. Gene list of the gene set, G14

Group ID	LOCUS	NAME	GO Biological Process	Function
	AT5G58870	FTSH PROTEASE 9, FTSH9	protein phosphorylation, proteolysis	ATP binding, ATP-dependent peptidase activity, ATPase activity, metalloendopeptidase activity, metallopeptidase activity, protein kinase activity, zinc ion binding
	AT5G50100	DCC1, DXXCXXC MOTIF 1	tissue regeneration	protein disulfide oxidoreductase activity
	AT5G10490	MSCS-LIKE 2, MSL2	chloroplast fission, ion transport, plastid organization, transmembrane transport	ion channel activity
	AT3G55610	DELTA 1-PYRROLINE-5-CARBOXYLATE SYNTHASE 2, P5CS2	L-proline biosynthetic process, embryo development ending in seed dormancy, hyperosmotic salinity response, oxidation-reduction process, phosphorylation, pollen development, proline biosynthetic process, response to abscisic acid	ATP binding, glutamate 5-kinase activity, glutamate-5-semialdehyde dehydrogenase activity
G14	AT1G70610	ABCB26, ATP-BINDING CASSETTE B26, ATTAP1, TAP1, TRANSPORTER ASSOCIATED WITH ANTIGEN PROCESSING PROTEIN 1	transmembrane transport	ATP binding, ATPase activity, coupled to transmembrane movement of substances, transporter activity
	AT5G67030	ABA1 (ABA DEFICIENT 1); zeaxanthin epoxidase	abscisic acid biosynthetic process, oxidation-reduction process, response to heat, response to osmotic stress, response to red light, response to water deprivation, sugar mediated signaling pathway, xanthophyll biosynthetic process	FAD binding, zeaxanthin epoxidase [overall] activity
	AT5G19850	hydrolase, alpha/beta fold family protein		hydrolase activity
	AT4G32770	ATSDX1, SUCROSE EXPORT DEFECTIVE 1, VITAMIN E DEFICIENT 1, VTE1	chlorophyll metabolic process, fatty acid metabolic process, phloem sucrose loading, regulation of defense response, response to high light intensity, response to oxidative stress, response to temperature stimulus, vitamin E biosynthetic process, xanthophyll metabolic process	isomerase activity, tocopherol cyclase activity
	AT1G17050	ATSPS2, SOLANESYL DIPHOSPHATE SYNTHASE 2, SPS2	isoprenoid biosynthetic process, photosynthesis, plastoquinone biosynthetic process	all-trans-nonaprenyl-diphosphate synthase (geranylgeranyl-diphosphate specific) activity, metal ion binding, trans-octaprenyltranstransferase activity

Supplemental Table S2. Gene list of the gene set, L09

Group ID	LOCUS	NAME	GO Biological Process	Function
G14	AT1G17745	3-PHOSPHOGLYCERATE DEHYDROGENASE, PGDH, PGDH2, PHOSPHOGLYCERATE DEHYDROGENASE 2	L-serine biosynthetic process, cellular amino acid metabolic process, oxidation-reduction process	protein binding
	AT5G13490	AAC2, ADP/ATP CARRIER 2	purine nucleotide transport, transmembrane transport	ATP:ADP antiporter activity
	AT5G25940	early nodulin-like protein;	unknown	molecular_function
	AT5G35360	ACETYL CO-ENZYME A CARBOXYLASE BIOTIN CARBOXYLASE SUBUNIT, CAC2	fatty acid biosynthetic process, malonyl-CoA biosynthetic process	ATP binding, acetyl-CoA carboxylase activity, biotin carboxylase activity, metal ion binding
	AT3G02360	6-PHOSPHOGLUCONATE DEHYDROGENASE 2, PGD2	D-gluconate metabolic process, male-female gamete recognition during double fertilization forming a zygote and endosperm, oxidation-reduction process, pentose-phosphate shunt, response to salt stress	NADP binding, phosphogluconate dehydrogenase (decarboxylating) activity
	AT3G17810	PYD1, PYRIMIDINE 1	de novo' pyrimidine nucleobase biosynthetic process, beta-alanine biosynthetic process, cellular response to nitrogen levels, oxidation-reduction process, thymine catabolic process, uracil catabolic process	NADH dehydrogenase activity, NADP binding, dihydropyrimidine dehydrogenase (NADP+) activity, dihydrouracil dehydrogenase (NAD+) activity, iron-sulfur cluster binding, oxidoreductase activity, acting on the CH-CH group of donors, protein homodimerization activity, uracil binding
	AT4G17530	ATRAB1C, ATRABD2C, RAB GTPASE HOMOLOG 1C, RAB1C	pollen tube growth	GTP binding, GTPase activity
